# The effect of social support, diabetes management self-efficacy, and diabetes distress on resilience among patients with type 2 diabetes: a moderated mediation analysis

**DOI:** 10.1186/s12889-024-18022-x

**Published:** 2024-02-15

**Authors:** Ali Mohammad Parviniannasab, Zohreh Faramarzian, Seyyed Ali Hosseini, Saeed Hamidizadeh, Mostafa Bijani

**Affiliations:** 1 Department of Nursing, School of Nursing, Larestan University of Medical Sciences, Larestan, Iran; 2 Nursing School, Larestan University of Medical Sciences, Larestan, Iran; 3 Department of Nursing, School of Nursing and Midwifery, Shiraz University of Medical Sciences, Shiraz, Iran; 4 Department of Medical Surgical Nursing, School of Nursing, Fasa University of Medical Sciences, Fasa, Iran

**Keywords:** Diabetes distress, Social support, Resilience, Self-efficacy, Self-management, Type 2 diabetes, Moderated mediation

## Abstract

**Background:**

Diabetes can result in distress. Improving Resilience is important in managing these conditions. It is also important to consider the mediating role of diabetes management self-efficacy (DMSE) between diabetes distress (DD) and Resilience. Likewise, understanding how social support (SS) buffers the impact of diabetes distress on Resilience is equally important.

**Methods:**

The present study used a cross-sectional design and included 403 participants diagnosed with type 2 diabetes (T2D). The study was conducted in the south of Iran. The participants were selected through convenience sampling from July 2022 to January 2023. Self-reported questionnaires, namely the Diabetes Distress Scale (DDS), Diabetes Management Self-Efficacy Scale (DMSE), Perceived Social Support Scale (PSSS), and Resilience Scale, were used for data collection in the present study. Structural equation modelling was used for moderated mediation analysis.

**Results:**

The results of the Pearson correlation analysis were indicative of a significant negative correlation (*p* < 0.01) between diabetes distress and diabetes management self-efficacy (r = − 0.607), social support (r = − 0.417), and Resilience (r = − 0.552). The findings further revealed that diabetes management self-efficacy had fully mediated the correlation between diabetes distress and Resilience. Moreover, the results indicated that social support had a moderating role in the DD-resilience link.

**Conclusions:**

The present study’s findings offer a new theoretical framework for T2DM that can benefit intervention designers. The results further suggest that promoting diabetes management self-efficacy can be an effective strategy to enhance Resilience and decrease diabetes distress. Also, nurses and other healthcare providers must pay close attention to support resources to improve the patients’ Resilience and evaluate the distress associated with diabetes.

## Background

Type 2 diabetes (T2D) can cause various macrovascular and microvascular physical complications [1]. In 2021, about 537 million people were diagnosed with diabetes. The number of diabetic patients is expected to rise to 643 million by 2030 and 783 million by 2045 [2]. Over 10 million Iranians have been diagnosed with diabetes [3]. Individuals living with diabetes encounter a range of challenges, including biopsychosocial issues [4, 5]. Diabetes can affect many organs of the body. It can increase the risk of complications such as retinopathy, nephropathy, neuropathy, and cardiovascular disease [6]. One of the significant causes of increased morbidity and mortality among individuals with diabetes is diabetes complications. Individuals with diabetes experience more depression than the general population (non-diabetic patients) [7].

Therefore, developing the ability to adapt and perform optimally to overcome emotional and psychological problems is vital for these patients [8]. This ability is explained through what is referred to as Resilience. The concept describes how some people can withstand difficulties and overcome problems [4]. Resilience is important in increasing psychological well-being and quality of life in stressful situations [5]. According to previous studies, both protective factors and several risk factors, such as diabetes distress (DD), can affect Resilience in patients with chronic diseases [9–11]. Distress is an important psychosocial factor that can adversely affect the health of individuals [12]. DD is a multidimensional/multifaceted construct that constitutes emotional burden, physician-related distress, regimen-related distress, and diabetes-related interpersonal distress [13]. Studies indicate that 44.6% of patients with diabetes worldwide suffer from high levels of DD [14]. Earlier studies have shown that patients’ Resilience decreased as DD increases [15, 16]. A protective factor of Resilience is self-efficacy.

According to the social learning theory, self-efficacy is understood as a person’s confidence in successfully performing certain activities [17]. The Diabetes Management Self-Efficacy (DMSE) scale was designed to measure the ability of individuals with diabetes to manage their condition effectively (their capacity to adhere to dietary and exercise regimens and medical treatment) [18]. Individuals with high levels of task-specific self-efficacy may be better prepared to handle stressful situations and cope effectively. A cross-sectional study has also pointed out that high self-efficacy is related to high resilience in patients with T2D [19]. A growing body of literature has shown that DD is associated with poorer self-efficacy [20]. Self-efficacy can reduce the adverse effects of distress and raise resilience [15]. Hence, self-efficacy can be considered as one of the components of Resilience and post-traumatic growth [21].

Further research is required to identify the psychosocial variables that can moderate the adverse effects of diabetes distress (DD) on other health factors. This is extremely important since it paves the way to identify effective strategies to improve Resilience among individuals with type 2 diabetes (T2D [22]. Social support (SS) is believed to be the moderating factor that can serve as a link to mitigate the negative impact of diabetes distress (DD) on other health factors, thereby enhancing Resilience among individuals with type 2 diabetes (T2D) [12]. According to the results of previous studies, social support plays a moderating role in diabetes distress and self-care behaviours [23], Glycemic Control [24], and task performance [25]. Social support can be defined as resources that other people (others) provide to help an individual cope with problems more effectively [26]. The Stress-Buffering Model argues that SS protects individuals against the effects of stress on their health and well-being [27]. Several studies have shown that patients who receive more social support display better adjustment and less emotional distress [23, 28]. According to the social support buffer model, individuals who receive little or no social support are more vulnerable to health-related stress. In contrast, those receiving more support can better tolerate these effects [12, 29].

**Fig. 1:**
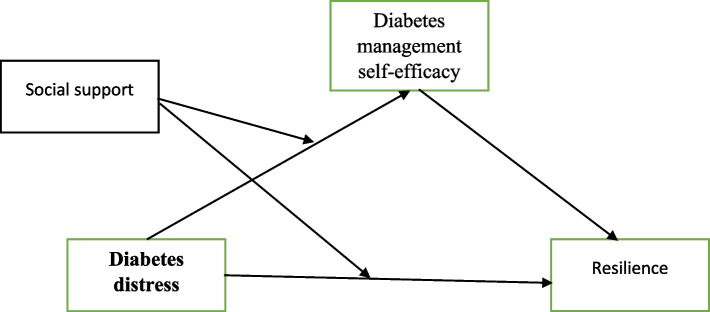
The proposed moderated mediation model

To the best of the authors’ knowledge, no studies have investigated the potential insulating effects of social support on resilience in individuals with type 2 diabetes (T2D). Whereas previous studies on the buffering hypothesis have compared individuals with and without chronic diseases, studies that have specifically examined diabetes distress among diabetic patients are missing. The evidence linking diabetes distress (DD) to resilience in individuals with type 2 diabetes (T2D) necessitates further research. In addition, previous studies [9, 11] have pointed to a significant positive correlation between social support (SS) and resilience. This study proposed a moderate mediation model to explore the probable link between DD, DMSE, SS, and Resilience. Based on the above discussions, the following hypotheses were proposed: (H1) the mediating role of DMSE between DD and Resilience; (H2) SS moderates the relationship between DD, DMSE and Resilience (Fig. 1).

## Methods

### Design and participants

A cross-sectional study was conducted on 403 patients diagnosed with type 2 diabetes (T2D). The participants were patients referred to Shahid Hashemi and Dalki clinics affiliated with Larestan and Shiraz University of Medical Sciences in Fars province in the south of Iran, respectively. Based on Wolf et al. [30], who state that 10 observations per estimated parameter are required to calculate the sample size for studies that use structural equation modelling, a minimum sample of 290 subjects was considered appropriate. To account for a 10% dropout rate, the minimum required sample size was determined to be 319 people.

Convenience sampling was used to select the participants. The inclusion criteria in the present study were 18 or over, holding a diabetes diagnosis certificate, and being literate. Non-volunteers were excluded from the study. Initially, 420 individuals signed the informed consent form and completed the questionnaire voluntarily. However, 17 questionnaires were excluded from further analysis because more than 20% of the information was missing.

### Data collection

From July 2022 to January 2023, team members possessing specific expertise in the investigated field handed out the questionnaires to the participants who met the defined criteria at the specified centre in a quiet room. Before distributing the questionnaires, the participants signed an informed consent and the importance of maintaining anonymity and confidentiality of their responses was highlighted. Each individual completed the questionnaire in a separate room to avoid their responses being influenced by others. While completing the questionnaire, the researchers would explain if there was any ambiguity in the questions. Completing the questionnaires took between 25 to 30 minutes. The participants returned their completed questionnaires on the same day.

### Measurements

The measurement tool initially comprised general demographic inquiries encompassing gender (male and female), age, education level, and marital status. Additionally, diabetes-related questions such as BMI, comorbidity, diabetes duration (years), and use of diabetes-related medications and four main scales measuring diabetes distress, diabetes management self-efficacy, social support, and resilience were followed. We used the tools with adequate reliability and validity, which are widely used.

### Resilience scale (RS)

The Resilience Scale was originally developed by Wagneild in 2009 [31]. The abridged version has been translated into Persian by Nourian (2015). The reliability and validity of the abridged version have been confirmed [32]. A confirmatory factor analysis (CFA) was run to check the scale’s validity in the present study. The CFA index of this scale indicated a good fit: *χ*2/df = 2.240, RMSEA = 0.057, CFI = 0.975, TLI = 0.966, and SRMR =0.031. The Resilience scale consists of 14 items, which are divided into three components: self-management (5 items), meaningfulness of life (5 items), and self-confidence (4 items). Responses are measured on a 5-point Likert scale ranging from 1 (completely disagree) to 5 (completely agree). Possible scores range from 14 to 60, with higher scores indicating greater levels of Resilience. The original Resilience Scale had a Cronbach’s α of 0.93 [31]. The reliability of the abridged Resilience Scale was 0.78 [32]. The Cronbach’s α values for the self-management, meaningfulness of life, self-confidence, and overall scales were 0.87, 0.89, 0.86, and 0.94, respectively.

### Diabetes management self-efficacy scale (DMSE)

The Diabetes Management Self-Efficacy Scale (DMSE) was developed by Bijl et al. in 1999 [33]. The Persian version of this scale has good reliability and validity [34]. A confirmatory factor analysis (CFA) was run to examine the validity of the construct in the present sample of diabetes patients. The index of CFA showed a good fit: *χ*2/df = 2.57, RMSEA = 0.065, CFI = 0.954, TLI = 0.942, and SRMR =0.062. The DMSE scale consists of 20 items with four domains: diet (9 items), monitoring (4 items), physical activity (4 items), and drug regimen (3 items). Respondents rate their level of agreement with each item using a 5-point Likert scale ranging from 1 (completely disagree) to 5 (completely agree). The possible score range for the DMSE is 20 to 100, with higher scores indicating higher levels of self-efficacy. The original version of the DMSE had a Cronbach’s α value of 0.81. In this study, Cronbach’s α value was 0.90, indicating a high level of internal consistency reliability for the scale.

### Diabetes distress scale (DDS)

The Diabetes Distress Scale (DDS) has been designed and evaluated by Polonsky in 2005 [35]. The scale and its Persian abridged version have been used in cross-cultural studies, and its reliability and validity have been confirmed [36]. In the current sample of diabetes patients, a confirmatory factor analysis (CFA) was performed to examine the validity of the construct. The CFA index showed a good fit: *χ*2/df = 2.98, RMSEA = 0.072, CFI = 0.960, TLI = 0.945, and SRMR =0.041. DDS consists of four subscales: emotional burden, physician-related distress, regimen-related distress, and diabetes-related interpersonal distress. Responses for each item were on a 6-point Likert scale range from 1 (no problem) to 6 (a severe problem; the scores on the scale range from 17 to 102. Higher scores reflect higher levels of self-efficacy. The original version of the DMSE scale has a Cronbach’s α value of 0.87% [35]. The Cronbach’s α value in the present study was 0.95.

### Perceived social support scale (PSSS)

The Perceived Social Support Scale (PSSS) was developed by Zimet et al. in 1988 [37]. The PSSS contains three domains (family, friends, and others) with 12 items and has acceptable reliability and validity [38]. However, a confirmatory factor analysis (CFA) was run in the present study to examine the scale’s validity. The index of CFA of this scale showed a good fit: *χ*2/df = 2.11, RMSEA = 0.054, CFI = 0.983, TLI = 0.972, and SRMR =0.035. Responses are measured on a 5-point Likert scale ranging from 0 (completely disagree) to 4 (completely agree). The scores on the PSSS range from 0 to 48, with higher scores indicating greater levels of perceived social support. In the original version of the PSSS scale, Cronbach’s α was 0.87 [37]. In the present study, the Cronbach’s α was 0.90.

### Statistical analysis

Descriptive statistics and correlation analysis were used to analyze the survey data. Also, a structural equation modelling (SEM) using IBM SPSS AMOS version 24 was used to examine the mediation role of diabetes management self-efficacy between diabetes distress and Resilience. Fit indices were examined using the χ2/df < 5 [39], goodness of fit index (GFI) > 0.90, Tucker-Lewis index (TLI) > 0.90, comparative fit index (CFI) > 0.90, the absolute index root mean square error of approximation (RMSEA) < 0.080, and standardized root mean square residual (SRMR) < 0.080 [40] to determine whether the assumed model conformed to the observed data. Sociodemographic and disease-related characteristics such as age, gender, BMI, and diabetes duration [12, 41, 42] were included as control variables in the data analysis. Finally, the moderator–mediator model with Hayes’s PROCESS macro was analyzed using SPSS v. 25 (Model 8) (2013) [43]. The 95% bootstrap confidence intervals (CI) were calculated using 5000 bootstrapped samples. CIs that did not contain 0 indicate a significant effect. The *P* values were set at *p* < 0.05 (two-tailed).

## Results

### Sample characteristics

The mean for the age of the participants in the present study was 46.29 ± 17.118 years. 76.9% of the participants were over 60 years old. 55.1% of the participants were female. 75.9% of the participants were married. 28.8% of them did not hold a high school diploma.

Additionally, 82.4% of the participants had been diagnosed with diabetes for less than 10 years. 46.2% of the participants were classified as overweight based on their body mass index. Most participants (49.4%) were on oral insulin treatment (see Table 1).

**Table 1:** Participant characteristics (*N* = 403)

*Characteristic*	Mean ± SD	Frequency	Percentage
Age (years)	46.29 ± 17.118		
< 60		310	76.9%
≥ 60		93	23.1%
**Gende**r			
Female		222	55.1%
Male		181	44.9%
**Education levels**			
Less than high school diploma		116	28.8%
High school diploma		88	21.8%
Academic		102	25.3%
Illiterate		97	24.1%
**Marital status**			
Single		97	24.1%
Married		306	75.9%
**Classification of BMI**			
Underweight		4	1%
Normal weight		186	46.1%
Overweight		208	52.9%
**Comorbidity**			
High blood pressure		101	25%
Arthritis		47	11.7%
Others		**100**	24.8%
No medical condition		155	38.5%
**Years of illness**			
0–10		322	82.4%
11–17		50	12.4%
> 18		21	5.2%
**Employment**			
Employed		53	13.2%
Unemployed		165	40.9%
Homemaker		185	45.9%
**Diabetes-Related Medication Use**			
Oral Meds		199	49.4%
Insulin		108	26.8%
Both		63	15.6%
None		33	8.2%

### Correlational findings

The results of the correlation analysis showed that DD was negatively and significantly related to Resilience (r = − 0.552, *p* < 0.01), DMSE (r = − 0.607, p < 0.01) and social support (r = − 0.417, *p* < 0.01). Additionally, DMSE (*r* = 0.743, *p* < 0.01) and social support (r = 0.611, *p* < 0.01) were positively and significantly related to Resilience (Table 2).

**Table 2:** Correlations, means and standards deviations of study variables

	1	2	3		4
1. DD	1				
2. SS	−0.417**	1			
3. resilience	−0.552**	0.611**	**1**		
4. DMSES	−0.607**	0.539*	0.743**	**1**	
M	37.73	31.95	**51.32**	**70.99**	
SD	16.97	8.39	**10.14**	**15.66**	

Using bivariate correlation analysis

*DD* Diabetes Distress, *DMSE* Diabetes Management Self-Efficacy, *Scale SS* Social Support

** *p* < 0.01

### Mediation analyses

Before data analysis, the data were checked for missing values, outliers, and normal distribution. The results of Little’s test indicated that the questionnaire data (Chi-Square = 2319.854, df = 2398, *p* = 0.871) were missing completely at random (MCAR). A non-significant result on Little’s test indicates no patterns in the missing data [44]. Mean imputation was used to replace the missing data. The study used skewness and kurtosis values with an accepted range of − 2 to + 2 (30) to test the normality. Confirmatory factor analysis was performed to assess the measurement model by examining the correlation between observed variables and latent constructs. The findings indicated that factor loading was above 0.50 in all measurement models, indicating a satisfactory factor loading [45].

**Fig. 2:**
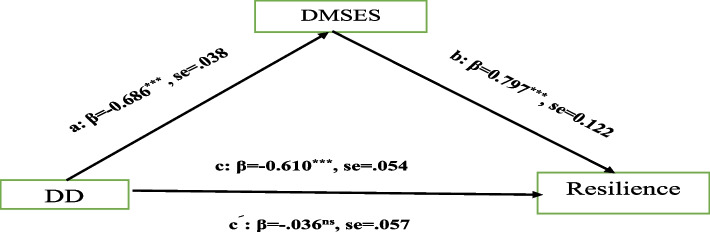
Hypothesized mediated model. Path c: total direct effect, path c’: direct effect. a: effects of DD on the mediator (DMSES); b—effects of the mediator on the Resilience. Using structural equation model (SEM)

The modified goodness of fit indexes of the SEM (χ2/df value of 4.75 (180.54/38 < 5), GFI = 0.923, AGFI = 0.901, CFI = 0.955, TLI = 0.936, NFI = 0.945, and RMSEA = 0.079) showed a good fit to the data. As shown in Table 3 and Fig. 2, the path of DD to Resilience as total effect (path cﹶ) was significant (t = − 12.15, β = − 0.610, *p* = 0.001). Bias-corrected bootstrap confidence intervals (CI) based on 5000 bootstrapping samples were used to evaluate the direct and indirect effects (28). Since the CI did not contain zeros, it was concluded that the correlation was significant. The path from DD to Resilience through DMSE was non-significant (path c^ﹶ^) (β = − 0.036, 95%CI: − 0.159, 0.067, *p* > 0.05). However, the indirect effect of DD on Resilience through DMSE was significant (β = − 0.546, 95%CI: − 0.650, − 0.0471, p = 0.001), indicating a full mediating effect of DMSE.

**Table 3:** Total, direct, and indirect effects of each path in this model using structural equation model

path	β	SE	BC 95% CI	
			Lower	Uppe
Total effect				
DD → Resilience	−0.610^***^	0.054	− 0.689	− 0.499
Direct effects				
DD → DMSES	−0.686^***^	0.038	−0.766	−0.577
DD → Resilience	− 0.036^ns^	0.057	− 0.159	0.067
Indirect effect				
DD → DMSES →Resilience	−0.546^***^	.045	−0.65	−0.0471

*SS* Social support, *DD* Diabetes distress

*N* = 403

*P < 0.05, **P < 0.01, ***P < 0.001

### Results of moderated mediation analysis

**Fig. 3:**
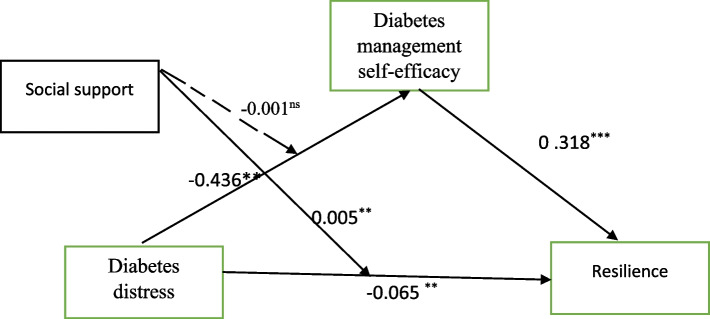
The moderated mediation model. ns, not significant; *p < 0.05, **p < 0.01, ***p < 0.001

**Fig. 4:**
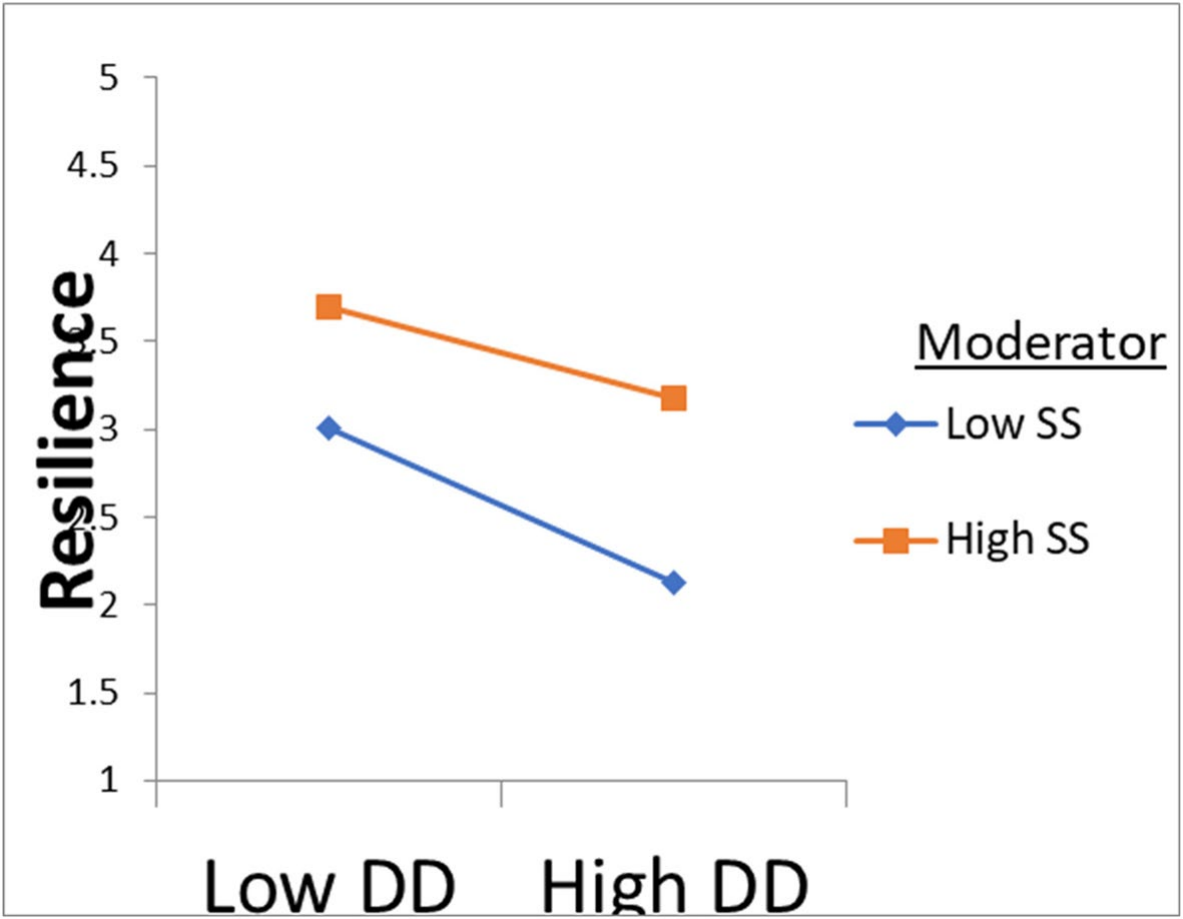
James Gaskin plot showing the interaction effect of DD and SS on resilience. SS dampens the negative relationship between DD and resilience. SS, social support; DD, diabetes distress

As shown in Table 4 and Fig. 3, Model 1 indicates that DD has had a significant effect on DMSE (*β* = − 0.436, *SE* = 0.039, 95%CI = [− 0.514, − 0.359]) and SS has had a non-moderating effect (*β* = − 0.001, *SE* = 0.003, 95%CI = [−.008, 0.005]). Model 2 indicates that DD has had a significant effect on Resilience (*β* = − 0.065, *SE* = 0.024, 95%CI = [− 0.114, − 0.017]) and SS has had a moderating effect (*β* = 0.005, *SE* = 0.002, 95%CI = [0.002, 0.009]). Based on these findings, it can be stated that hypothesis 2 is partially supported. In addition, a simple slope test was utilized to exhibit the significant interaction at 1 SD below the mean and 1 SD above the mean for social support. The results suggest that a higher level of SS diminishes the effect of diabetes distress on resilience via diabetes management self-efficacy (See Fig. 4).

**Table 4:** Results of the moderated mediation model analysis

variable	Model 1(DMSE)					Model 2(Resilience)				
	β	SE	t	*P*	95%*CI*	β	SE	t	*P*	95%*CI*
Control variables										
Age	−3.4193	1.226	2.788	0.005 ^**^	−5.830, 1.008	−0.835	0.673	−1.240	0.215	−2.159, 0.488
Gender	0.937	1.143	0.819	0.413	− 1.311, 3.186	0.672	0.622	1.079	0.280	−0.551, 1.896
BMI	−0.927	1.164	− 0.796	0.426	−3.217, 1.362	−0.207	0.633	− 0.326	0.744	−1.453, 1.039
diabetes duration	9.716	11.488	0.845	0.398	−12.870,32.302	8.864	6.253	1.417	0.157	−3.429, 21.15
DD	−0.436	0.039	−11.038	0.000^***^	−0.514, − 0.359	− 0.065	0.024	−2.671	0.007	−0.114,-0.017
DMSE						0 .318	0.027	11.64	0.000^***^	0.264, 0.372
SS	0.670	0.077	8.667	0.000**	0.518, 0.823	0.342	0.045	7.456	0.000^***^	0.252, 0.432
DD x SS	−0.001	0.003	− 0.371	0.710	−.008, 0.005	0.005	0.002	2.978	0.003**	0.002, 0.009
R^2^			0.470					0.789		
F			50.145						81.649	

Using Hayes’ (2013) PROCESS macro (Model 8) in the SPSS

*BMI* Body mass index, *DD* Diabetes distress, *SS* Social support, *CI* Confidence interval, *DMSE* Diabetes management self-efficacy

N = 834

*p<0.05, **p<0.01, ***p<0.001

## Discussion

Hypothesis 1, which states that DMSE plays a mediating role between DD and Resilience, was confirmed based on the findings of the present study. The effect of DD on the Resilience of patients with diabetes is understood through a direct path and an indirect path (through influencing DMSE). The results of the present study showed that DD had a negative effect on resilience. This is consistent with the findings of earlier research, which indicate that higher levels of psychological distress are associated with lower levels of resilience [12]. In different situations or when being exposed to risk factors, resilience helps patients solve their problems and cope better [46]. Resilience theory highlights that stressful conditions can decrease an individual’s Resilience, which may, in turn, jeopardize his/her physical and mental health [47]. This can be further suggestive that the negative impact of diabetes distress should be taken into consideration before designing interventions to increase resilience in diabetic patients. In the present study, DD had a negative and significant impact on DMSE. These findings are consistent with the findings of previous studies that show that diabetic patients who experience severe complications are more likely to experience distress and exhibit lower levels of self-care and self-efficacy [48, 49]. Also, the results of earlier studies indicate that high diabetes distress can impede patients from displaying/performing self-management behaviours [20, 50]. The psychological distress of diabetes can significantly affect self-management in diabetic patients. Therefore, health professionals must take practical steps to identify these conditions and understand the effect of diabetes distress on the health outcomes of diabetics. Research suggests that higher levels of self-efficacy in diabetic patients can enhance their resistance to the adverse effects of diabetes distress, leading to a possible reduction in symptoms [22]. Therefore, DD affects patients’ resilience directly and indirectly. The findings of another study have shown that self-efficacy can mediate the correlation between negative emotional regulation and resilience in diabetic patients [51]. It is suggested that diabetic patients may enhance their Resilience by employing positive emotion regulation strategies, which can be facilitated by diabetes self-efficacy.

The findings related to hypothesis 2 showed that social support buffered the relationship between DD and resilience. The present study findings further point out that increased social support buffers or reduces the adverse effects of DD on resilience in patients with T2D. These findings are consistent with previous studies on chronic illnesses such as cancer and cardiovascular disease, which have similarly revealed that increased social support can buffer or reduce the adverse effects of psychological distress on resilience [29, 52]. Several studies have shown that social support can buffer the adverse effects of diabetes distress on various health outcomes. For instance, social support has been found to lessen the link between diabetes distress and depressive symptoms [12], quality of life [23], and glycemic control [24]. The results of the present study suggest that sources of social support are essential in helping patients with T2D manage the effects of DD on their health. According to the buffer-stress model, perceived social support affects a person’s health because it protects him/her from the adverse effects of excessive stress [53]. Therefore, alleviating the adverse effects of DD through such resources may be beneficial for improving resilience. The findings from these studies indicate that enhanced social support can increase the psychological resilience of adults with diabetes. According to the present study findings, these strategies may be especially beneficial for patients experiencing diabetes distress. The present study intended to investigate the mediators and moderators between DD and resilience in patients with T2D, providing valuable insights that can serve as a framework for counselling to decrease DD, emphasizing the role of social support and self-efficacy.

### Limitations

Despite its contributions, this study has some limitations that should be considered when interpreting the results. Firstly, the present study is cross-sectional, and the SEM approach only shows associations between the included variables, not causality. Future longitudinal or experimental studies are required to identify causality between the variables. Secondly, the study focused only on social support, diabetes distress, and diabetes management self-efficacy as factors influencing Resilience. Other psychosocial and physiological factors contributing to the improvement of Resilience should be the topic of future inquiry. Finally, this study relied on self-report measures for data collection, and the participants’ subjective views might influence the results.

### Clinical implications

Given the direct and indirect effects of DD, social support, and self-efficacy on Resilience, interventions aiming at promoting Resilience in patients with diabetes should focus on simultaneously increasing social support and self-efficacy to reduce diabetes-related distress. Improved self-efficacy can enhance Resilience, leading to possible improvements in self-management behaviour (However, it may not be beneficial for Resilience in individuals with high levels of diabetes distress). Therefore, healthcare providers, such as nurses, should prioritize assessing and addressing diabetes distress before intervening to improve self-efficacy and Resilience.

## Conclusion

This study provides preliminary evidence of the mediating role of self-efficacy between DD and Resilience. Healthcare providers must prioritize interventions that promote self-efficacy and enhance social support to reduce DD and improve Resilience in patients with diabetes. Furthermore, given the significant impact of social support in reducing DD, nurses and other healthcare providers should pay specific attention to support the resources and evaluate the distress related to diabetes to improve the patients’resilience.

## Data Availability

The datasets generated and/or analysed during the current study are not publicly available due to the necessity to ensure participant confidentiality policies and laws of the country but are available from the corresponding author on reasonable request.

## Abbreviations

DMSE: diabetes management self-efficacy
DD: diabetes distress
SS: social support
T2D: type 2 diabetes
PSSS: Perceived Social Support Scale
RS: Resilience scale
SEM: structural equation modeling
GFI: goodness of fit index
NFI: normed fit index
IFI: incremental fit index
TLI: Tucker-Lewis index
CFI: comparative fit index
RMSEA: absolute index root mean square error of approximation
MCAR: missing completely at random
